# Long-term weight-change slope, weight fluctuation and risk of type 2 diabetes mellitus in middle-aged Japanese men and women: findings of Aichi Workers' Cohort Study

**DOI:** 10.1038/nutd.2017.5

**Published:** 2017-03-20

**Authors:** Y Zhang, H Yatsuya, Y Li, C Chiang, Y Hirakawa, N Kawazoe, K Tamakoshi, H Toyoshima, A Aoyama

**Affiliations:** 1 Department of Public Health and Health Systems, Nagoya University Graduate School of Medicine, Nagoya, Japan; 2 Department of Public Health, Fujita Health University School of Medicine, Toyoake, Aichi, Japan; 3 Department of Nursing, Nagoya University Graduate School of Medicine, Nagoya, Japan; 4 Education and Clinical Research Training Centre, Anjo Kosei Hospital, Anjo, Aichi, Japan

## Abstract

**Objective:**

This study aims to investigate the association of long-term weight-change slopes, weight fluctuation and the risk of type 2 diabetes mellitus (T2DM) in middle-aged Japanese men and women.

**Methods:**

A total of 4234 participants of Aichi Workers' Cohort Study who were aged 35–66 years and free of diabetes in 2002 were followed through 2014. Past body weights at the ages of 20, 25, 30, 40 years, and 5 years before baseline as well as measured body weight at baseline were regressed on the ages. Slope and root-mean-square-error of the regression line were obtained and used to represent the weight changes and the weight fluctuation, respectively. The associations of the weight-change slopes and the weight fluctuation with incident T2DM were estimated by Cox proportional hazards models.

**Results:**

During the median follow-up of 12.2 years, 400 incident cases of T2DM were documented. After adjustment for baseline overweight and other lifestyle covariates, the weight-change slopes were significantly associated with higher incidence of T2DM (hazard ratio (HR): 1.80, 95% confident interval (CI): 1.17–2.77 for men; and HR: 2.78, 95% CI: 1.07–7.23 for women), while the weight fluctuation was not (HR: 1.08, 95% CI: 1.00–1.18 for men and HR: 1.02, 95% CI: 0.84–1.25 for women).

**Conclusions:**

Regardless of the presence of overweight, the long-term weight-change slopes were significantly associated with the increased risk of T2DM; however, the weight fluctuation was not associated with the risk of T2DM in middle-aged Japanese men and women.

## Introduction

Type 2 diabetes mellitus (T2DM) is a major risk factor for cardiovascular diseases as well as is a non-communicable disease with disabling and life-threatening complications. It is increasingly imposing a large economic burden on the individuals and healthcare systems all over the world.^1^ Indeed, age-adjusted T2DM prevalence in Japan was reported as 7.9% in 2010 among the total population, and this rate is expected to rise to 9.8% by 2030.^2^ Among the many established and possible risk factors of T2DM, excessive body weight is a major one,^3, 4^ indicating that weight management is a key to prevent T2DM.^5^

Previous studies have reported that long-term weight change since early adulthood was associated with the risk of T2DM.^6, 7, 8^ In those studies, weight changes were typically defined as the difference in weights of two time points. However, the same weight gain occurred in different length of time may have different health consequences. Weight-change slopes defined as kg per year, that is, average annual speed of weight changes could provide such information. Furthermore, weight fluctuation, the magnitude and the number of weight loss and gain, has reportedly been associated with worse cardio-metabolic indicators.^9, 10, 11, 12^ However, only a few studies prospectively examined the relation between these weight-change measures and incident T2DM,^13, 14^ and none of them has been among any Asian population.

Therefore, the objective of this study was to assess the long-term weight-change slopes and the weight fluctuation in relation to the risk of T2DM incidence among middle-aged Japanese men and women independent of current overweight or current BMI. For the participants aged 45 or over, we have also performed the analyses that use weight-change slope and fluctuation up to middle age (40 years old). Hypothesized positive association observed between these measures and T2DM incidence could be a basis for promoting more prevention effort against positive weight-change slope before becoming middle age, only at which most preventive health services start.

## Materials and methods

### The Aichi Workers' Cohort Study

The Aichi Workers' Cohort Study is an ongoing study on diabetes and cardiovascular disease epidemiology. In 2002, we enrolled 6648 Japanese civil servants aged 35–66 years who provided written informed consent and responded to self-reported questionnaire about their lifestyle. The study protocol was approved by the Ethics Review Committee of Nagoya University School of Medicine, Nagoya, Japan (approval number: 504-4).

For the present analysis, we excluded the following participants: (1) those who did not agree to provide their medical history or annual health check-up data (*n*=1111); (2) those who had T2DM defined as self-reported medication use or baseline blood glucose level >126 mg dl^−1^ (*n*=497); (3) those who reported to have any cancer (*n*=36); (4) those who did not have the data of baseline body weight (*n*=31) and body weight at the age of 20 (*n*=271); (5) those who had only two or less body weight measures (*n*=5) or (6) those who did not have the data of baseline blood glucose level (*n*=73), or other covariates (*n*=390). Finally, 4234 participants (3317 men and 917 women) were included in the present study.

### Body weight data and definition of the weight-change slope and the weight fluctuation

Baseline weight and height were measured in the annual health check-up. Height was measured in the standing position to the nearest 0.1 cm, and body weight to the nearest 0.1 kg. Past weights at the ages of 20, 25, 30, 40 years, and 5 years before baseline (weight in 1997) were self-reported in the questionnaire depending on the age of the participants at baseline. These weights constituted the body weight data set over time at five (for participant 40 years old or younger) or six ages (for participants over 40 years old). The body weights were regressed on age to obtain the age-weight regression line. For the participants who missed 1–3 data of body weights, the regression lines were created based on 3–5 body weight data. The slope of this regression line represents the overall trend of body weight change, thus, it was used as the index of the long-term body weight change in the present study.

The root-mean-square-error (RMSE) is a measure of average deviation in weight from the age-weight regression line,^9, 10, 11, 13, 14, 15^ and used as the indicator of weight fluctuation in the present study. As RMSE and slope of the linear regression line were not correlated (Pearson correlation coefficient=0.153), RMSE was considered as a measure of variability in weight independent of the overall trend.

For those aged 45 or older at baseline, we have also calculated weight-change slopes and fluctuation using only weights up to middle age (40 years old) in order to examine the associations of the weight-change measures before middle age with T2DM incidence.

### Definition of covariates

Potential confounders were statistically adjusted for as covariates. Smoking status was classified into three categories (never, past and current). Alcohol drinking habit was assessed as the frequency per week, which was categorized into three groups (<1, 1–4 and 5–7 occasions per week). Leisure-time physical activity was assessed by asking question ‘Are you engaged in any physical activity for a total of at least 60 min for more than a day per month?' We then classified the participants into two categories (yes, no). Self-reported usual sleep duration^16^ was classified into two groups (<7, ⩾7 h per day). The presence of family history of diabetes was defined as one among first-degree relatives (parents or siblings) (yes or no). Everyday breakfast eating habit^17^ was dichotomized into two categories (yes, no).

Fasting blood glucose (FBG) was enzymatically determined by the hexokinase method using serum. FBG was classified into two categories (⩾100 mg dl^−1^, <100 mg dl^−1^).^18^ BMI was computed as body weight (kg) divided by the square of height (m). In the present study, overweight was defined as BMI 25 kg m^−2^ or greater.^19, 20^

### Ascertainment of incident T2DM

The participants were followed through 31 December 2014. The person-years were calculated from baseline to the date of censoring, ascertainment of incident T2DM, or the end of the follow-up, whichever came first. Participants were censored when they died or retired from the workplace except for those who agreed to provide their health history information to the researchers after their retirement. We ascertained incident T2DM cases through annual mandatory health check-ups at work places until retirement and questionnaire surveys during employment as well as after retirement. For the former, the health check-up data were annually reviewed and the T2DM incidence was defined when the FBG level first became 126 mg dl^−1^ or over or hemoglobin A1c (HbA1c) became 6.5% or over (with US National Glycohemoglobin Standardization Program method). HbA1c test was provided only to the employees aged 40, 45, 50 and 55 years old until 2007, and to those with positive urinary glucose after 2008. Self-administrated questionnaire survey was carried out approximately biennial between 2004 and 2014. Participants were asked to report their medical histories of selected conditions, including T2DM. The participants who reported T2DM history were requested to provide the detailed contact information of the physicians who took charge of their disease management. We confirmed the participants' medical records with their physicians, if written consents from the participants were obtained. Briefly, of the 400 incident T2DM cases in the present study, 147 self-reported the incidence, which were confirmed either by their physician (*n*=56) or blood test at the annual health check-up (*n*=100, because of overlap adding these numbers is not equal to 147). Although there were cases only with self-report (*n*=30, 20% of 147 self-reports), mainly due to their not having provided consent for our physician survey, our previous validation study proved the high accuracy of self-reports (95%) by reviewing the participants' self-reports and medical records.^21^

### Statistical analysis

All the analyses were carried out stratified by sex. Cox proportional hazards regression models were used to determine the hazard ratios (HRs) and respective 95% confidence intervals (CIs) of the association of the weight-change slopes and the weight fluctuation with the incidence of T2DM adjusting for potential confounding factors. The Model 1 included age, smoking status and alcohol drinking frequency, leisure-time physical activity, everyday eating of breakfast, sleep duration, family history of diabetes and FBG level at baseline. Model 2 further included the presence of overweight at baseline. A model adjusting for baseline BMI (continuous) was also performed. The weight-change slope and the RMSE values were simultaneously entered in the models. We have also carried out analyses using weight-change slope and the RMSE constructed with weights up to middle age for those aged 45 or over. Furthermore, additional analyses were carried out by restricting to never-smoking men and by stratifying participants into positive and negative slopes (for the RMSE analysis).

Sensitivity analysis was conducted by excluding the incident cases of T2DM that developed the disease within 3 years from baseline (*n*=150) as the possible pre-clinical diabetic conditions could have some influence on one's baseline weight. The second sensitivity analysis was carried out by excluding participants who self-reported histories of dieting (*n*=263) because weight regain frequently occurs after intentional weight loss^22, 23^ and this weight cycling may have distorted estimation of the weight-change slopes and the fluctuation. The third sensitivity analysis was performed by excluding the incident T2DM cases with only self-report (*n*=30).

All the analyses were carried out with IBM SPSS Statistics version 23.0 for Windows (IBM Corporation, Armonk, NY, USA). All the tests were two-sided and the significance level was set at *P*<0.05.

## Results

The mean age of men and women were 47.6 and 46.2 years old, respectively. The prevalence of current smoking was 34.3% in men and 6.7% in women (Table 1). Family history of diabetes was reported from 14.0% of men and 19.3% of women. The proportion of participants whose FBG levels were 100 mg dl^−1^ or higher was 19.8% in men and 11.7% in women. On average, men gained 0.30 kg per year and women gained 0.14 kg per year from age 20. The mean RMSE was 1.60 kg in men and 1.34 kg in women.

During a median 12.2 years of follow-up, 400 incident cases of T2DM were documented (330 men and 70 women). The crude incident rates of T2DM were 10.1 per 1000 person-years among men and 8.3 per 1000 person-years among women. After adjustment for the Model 1 covariates, the weight-change slope per 1 kg per year increase was significantly associated with higher incidence of T2DM (HR: 2.55, 95% CI: 1.72–3.78 for men; and HR: 5.60, 95% CI: 2.48–12.62 for women) (Table 2). After further adjustment for baseline overweight (Model 2), the HRs of weight-change slopes with T2DM incidence were attenuated but remained statistically significant in both men and women (HR: 1.80, 95% CI: 1.17–2.77 for men and HR: 2.78, 95% CI: 1.07–7.23 for women). The analyses adjusting for continuous BMI yielded similar results in men (HR: 1.69, 95% CI: 1.08–2.67), however, although the HR estimate was similar to that of men, the association was significantly attenuated in women (HR: 1.67, 95% CI: 0.54–5.13). The RMSE was positively associated with the incidence of T2DM in men (HR: 1.09, 95% CI: 1.00–1.19) but not in women (HR: 1.03, 95% CI: 0.85–1.24) in Model 1. The associations changed little in men and women after further adjustment for baseline overweight (HR: 1.08, 95% CI: 1.00–1.18 for men; and HR: 1.02, 95% CI: 0.84–1.25 for women). The RMSE was not significantly associated with incident T2DM in men and women in the analyses adjusting for continuous BMI (Model 2 HR: 1.07, 95% CI: 0.99–1.17 for men and Model 2 HR 1.03, 95% CI: 0.84–1.26 for women).

The association of weight-change slope and T2DM incidence became stronger in never-smoking men (Model 2 HR: 2.69, 95% CI: 1.39–5.21). However, RMSE was not related to T2DM incidence in never-smoking men (Model 2 HR: 1.16, 95% CI: 0.99–1.35).

The RMSE was significantly associated with higher T2DM incidence among men with positive slopes (Model 2 HR: 1.11, 95% CI: 1.02–1.21), but not in men with negative slopes (Model 2 HR: 1.16, 95% CI: 0.88–1.53) as well as in women with positive and negative slopes (Model 2 HR: 1.11, 95% CI: 0.91–1.37; and Model 2 HR: 0.83, 95% CI: 0.44–1.56, respectively).

The associations of weight-change slope up to middle age for those aged 45 or over with T2DM incidence seemed similar to those of the main analysis in both men and women (Model 2 HR adjusted for overweight: 2.18, 95% CI: 1.32–3.59 and Model 2 HR adjusted for overweight: 3.66, 95% CI: 1.32–10.17, respectively) (Table 3). RMSE up to middle age was not related to T2DM incidence in either sex (Model 2 HR adjusted for overweight: 0.96, 95% CI: 0.84–1.10 for men and Model 2 HR adjusted for overweight: 1.01, 95% CI: 0.72–1.41 for women).

Excluding incident cases whose follow-up period were <3 years slightly attenuated the association of weight-change slopes with T2DM incidence in men (Model 2 HR: 1.66, 95% CI: 0.98–2.80) but not in women (Model 2 HR: 4.06, 95% CI: 1.19–13.92). The association of the weight fluctuation with T2DM incidence did not change in both men and women (Model 2 HR: 1.09, 95% CI: 0.98–1.21 for men and Model 2 HR: 0.97, 95% CI: 0.74–1.29 for women). The analyses that excluded participants with experiences of dieting yielded similar results for weight-change slopes (Model 2 HR: 1.58, 95% CI: 0.98–2.55 for men and Model 2 HR: 3.28, 95% CI: 1.16–9.30 for women). In contrast, the weight fluctuation was significantly associated with the elevated risk of T2DM in men (Model 2 HR: 1.11, 95% CI: 1.01–1.21) but not in women (Model 2 HR: 0.98, 95% CI: 0.77–1.25). The analysis that excluded the only self-report cases (*n*=30) yielded the similar result with the main analysis. The Model 2 HRs of the weight-change slopes with T2DM incidence were 1.85 (95% CI: 1.18–2.90) in men and 2.79 (95% CI: 1.06–7.33) in women. The RMSE was positively associated with the incidence of T2DM in men (Model 2 HR: 1.09, 95% CI: 1.00–1.19) but not in women (Model 2 HR: 1.03, 95% CI: 0.84–1.26).

## Discussion

To our knowledge, this is the first study to explore the association between the weight-change slope, the weight fluctuation since early adulthood, and the T2DM incidence among Japanese population. In the present study, we regressed weights since 20 years of age to baseline on ages, and employed the slope and RMSE of the regression line to represent the long-term overall weight-change trends and the fluctuation. This study showed significant positive associations between the long-term weight-change slopes and subsequent risk of T2DM after adjustment for many possible potential confounding variables, including baseline overweight, FBG and lifestyle factors. However, we did not find the significant association between the weight fluctuation and risk of T2DM in the current study.

Epidemiological studies focusing on the long-term weight-change trends are relatively rare. Previous studies reported the associations of weight change (difference in weights at two time points since early adulthood) with T2DM risk. For example, compared to the participants with relatively stable weight, weight gain of 2.5 kg or more was associated with the increased risk of T2DM in a large-scale cohort study of American men.^6^ Weight gains of 5 kg for men and 10 kg for women were significantly associated with the elevated T2DM risk in the Japan Public Health Center-based Prospective Study.^8^

Instead of using difference in weights at only two time points, we regressed weights on ages in linear model for each participant, and we employed the slope of the regression line to represent the long-term tendency of weight gain and loss in the present study. We found that weight-change slope since early adulthood was associated with the elevated risk of T2DM independent of the presence of baseline overweight. One of the possible mechanisms would be the altered adipose tissue distribution and resulting higher insulin concentration associated with more rapid weight gain.^15^ Moreover, although we have statistically adjusted for leisure-time physical activity, physical inactivity may be related to the present finding.^24, 25^

The association of the weight-change slope and incident T2DM seemed stronger in women. This would likely to be explained by the higher prevalence of smoking in men than in women (34.3 vs 6.7%) as the analysis restricted to never-smoking men yielded result similar to women.^26^

In the present study, we did not find significant associations between the long-term weight fluctuation and T2DM risk in both sexes. However, additional analyses revealed that, in men with positive slope, the RMSE was significantly associated with T2DM incidence. The latter findings could be in line with previous epidemiological studies that found its associations with metabolic disorders.^9, 10, 11^ Also, French and colleagues found the positive association between the long-term weight fluctuation and T2DM in American old women.^13^ It would be important to keep in mind that the degree of RMSE in the current study was much lower than the study conducted by French and colleagues. The mean values of RMSE across quartiles were 0.9, 2.0, 3.4 and 7.4, respectively in French and colleagues' study, while the corresponding values were 0.5, 1.0, 1.6, and 3.1, respectively in our study. However, another cohort study conducted in the United States, which also had large RMSE values, did not find association of weight fluctuation with T2DM.^15^ Nevertheless, further studies are needed to examine the association of the long-term weight fluctuation and incident T2DM.

The strengths of our study include the prospective cohort design, with up to 12-year follow-up for the incident T2DM, and with men and women included in the sample. However, our study also has several limitations that need to be considered. First, past weights at the ages of 20, 25, 30, 40 years, and 5 years before baseline were recalled and self-reported. However, a previous study indicated the good accuracy of recalled past body weights over a long period among middle-aged Japanese men.^27^ Second, the participants who had the large weight-change slope and RMSE since early adulthood might have developed T2DM before baseline, thus they were more likely to be excluded for the current study. This might lead to underestimation of the association between the weight-change slope, the RMSE and incident T2DM. Third, T2DM was ascertained using FBG in the health check-up as HbA1c was not uniformly obtained. A previous Japanese study reported that criterion using HbA1c identified more T2DM cases than the one using FBG.^28^ Furthermore, lower HbA1c cutoff values were reported in Chinese and Japanese studies^29, 30^ to yield higher sensitivity with similar level of specificity compared with the cutoff of 6.5%. In the present study, 153 T2DM cases were identified before 2008 and had both FBG and HbA1c values. Similar to the previous reports, HbA1c criterion was met in more cases (*n*=108) than FBG criterion (*n*=66) among the 153 cases (21 fulfilled both criteria). These findings as well as the fact that only self-reports were used after retirement suggest that more T2DM cases remained unidentified in non-cases, which implies underestimation of the true association in the present study. Fourth, RMSE, which was used as the indicator of weight fluctuation, may not be appropriate for those who had non-linear body weight-change curve.^15^ Lastly, we only assessed the long-term weight-change slopes and weight fluctuation before baseline in relation to the T2DM in the present study. Future study should be conducted to compare the contribution of weight-change slopes and weight fluctuation before and after baseline to T2DM incidence.

In conclusion, the present study revealed that the long-term weight-change slopes were significantly associated with the increased risk of T2DM independent of the presence of overweight, FBG level, as well as the other lifestyle risk factors at baseline. Our finding suggests that weight control starting from early adulthood may be effective for reducing the risk of T2DM in middle-aged Japanese men and women.

## Figures and Tables

**Table 1:** Baseline characteristics of the participants of Aichi Workers' Cohort Study, stratified by sex

*Characteristics*	*Men (*n=*3317)*	*Women (*n=*917)*
*Age (%)*		
<50 years	56.0	63.7
⩾50 years	44.0	36.3

*Smoking status (%)*		
Never smoker	37.6	88.8
Former smoker	28.0	4.6
Current smoker	34.3	6.7

*Alcohol drinking (%)*		
<1 occasion per week	26.6	56.8
1–4 occasions per week	25.8	24.4
5–7 occasion per week	47.6	18.8

*Breakfast eating (%)*		
Everyday eater	80.5	74.3
Non-everyday eater	19.5	25.7

*Sleep duration (%)*		
⩾7 h per day	48.0	35.0
<7 h per day	52.0	65.0

*Regular physical activity (%)*		
Yes	59.0	44.8
No	41.0	55.2

*Family history of diabetes (%)*		
No	86.0	80.7
Yes	14.0	19.3

*Fasting blood glucose (%)*		
<100 mg dl^*−*1^	80.2	88.3
⩾100 mg dl^−1^	19.8	11.7

*Baseline overweight (%)*		
No (BMI<25)	76.4	88.9
Yes (BMI⩾25)	23.6	11.1
Weight-change slope (kg per year)	0.30 (0.28)	0.14 (0.27)
Weight fluctuation (kg)	1.60 (1.21)	1.33 (1.07)

Abbreviation: BMI, body mass index.

The Data was shown as percentages for categorical variables and mean (s.d.) for continuous variables.

**Table 2:** Multivariate-adjusted hazard ratios and the 95% confidence intervals for predicting the effect of weight-change slope and RMSE on the incident type 2 diabetes, among the participants of Aichi Workers' Cohort Study, 2002–2014

	*Men*	*Women*
Number of incident cases	330	70
Total number of participants	3317	917
Person years	32695.8	8408.3
Crude incidence (1/1000 person-years)	10.1	8.3

*Hazard ratio (95% CI)*		
Slope		
Model 1^a^	2.55 (1.72–3.78)	5.60 (2.48–12.62)
Model 2^b^	1.80 (1.17–2.77)	2.78 (1.07–7.23)
RMSE		
Model 1^a^	1.09 (1.00–1.19)	1.03 (0.85–1.24)
Model 2^b^	1.08 (1.00–1.18)	1.02 (0.84–1.25)

Abbreviations: CI, confidence intervals; RMSE: root-mean-square-error.

a Model 1 included slope (continuous), RMSE (continuous), age (<50 and ⩾50 years old), smoking status (never, former and current), drinking frequency (<1, 1–4 and 5–7 occasions per week), leisure-time physical activity (yes, no), everyday eating of breakfast (yes, no), usual sleep duration (⩾7, <7 h per day), family history of diabetes (yes, no) and FBG level at baseline (<100 mg dl^−1^, ⩾100 mg dl^−1^).

b Model 2 included variables in the model 1 plus baseline overweight (baseline BMI <25, baseline BMI ⩾25).

**Table 3:** Multivariate-adjusted hazard ratios and the 95% confidence intervals for predicting the effect of weight-change slope and RMSE up to middle age on the incident type 2 diabetes, among the participants aged 45 and over of Aichi Workers' Cohort Study, 2002–2014

	*Men*	*Women*
*Hazard ratio (95% CI)*		
Slope		
Model 1^a^	2.70 (1.69–4.32)	5.91 (2.31–15.09)
Model 2^b^	2.18 (1.32–3.59)	3.66 (1.32–10.17)
RMSE^a^		
Model 1^b^	0.96 (0.83–1.10)	1.04 (0.75–1.45)
Model 2^b^	0.96 (0.84–1.10)	1.01 (0.72–1.41)

Abbreviations: CI, confidence intervals; RMSE, root-mean-square-error.

a Model 1 included slope (continuous), RMSE (continuous), age (<50 and ⩾50 years old), smoking status (never, former and current), drinking frequency (<1, 1–4 and 5–7 occasions per week), leisure-time physical activity (yes, no), everyday eating of breakfast (yes, no), usual sleep duration (⩾7, <7 h per day), family history of diabetes (yes, no) and FBG level at baseline (<100 mg dl^−1^, ⩾100 mg dl^−1^).

b Model 2 included variables in the model 1 plus baseline overweight (baseline BMI <25, baseline BMI ⩾25).
